# SCSilicon: a tool for synthetic single-cell DNA sequencing data generation

**DOI:** 10.1186/s12864-022-08566-w

**Published:** 2022-05-11

**Authors:** Xikang Feng, Lingxi Chen

**Affiliations:** 1 School of Software, Northwestern Polytechnical University, Xi’an, Shaanxi, 710072 China; 2 Department of Computer Science, City University of Hong Kong, Tat Chee Avenue, Kowloon, Hong Kong, China

**Keywords:** Single-cell sequencing, Simulation, Copy number variation

## Abstract

**Background:**

Single-cell DNA sequencing is getting indispensable in the study of cell-specific cancer genomics. The performance of computational tools that tackle single-cell genome aberrations may be nevertheless undervalued or overvalued, owing to the insufficient size of benchmarking data. *In silicon* simulation is a cost-effective approach to generate as many single-cell genomes as possible in a controlled manner to make reliable and valid benchmarking.

**Results:**

This study proposes a new tool, SCSilicon, which efficiently generates single-cell *in silicon* DNA reads with minimum manual intervention. SCSilicon automatically creates a set of genomic aberrations, including SNP, SNV, Indel, and CNV. Besides, SCSilicon yields the ground truth of CNV segmentation breakpoints and subclone cell labels. We have manually inspected a series of synthetic variations. We conducted a sanity check of the start-of-the-art single-cell CNV callers and found SCYN was the most robust one.

**Conclusions:**

SCSilicon is a user-friendly software package for users to develop and benchmark single-cell CNV callers. Source code of SCSilicon is available at https://github.com/xikanfeng2/SCSilicon.

**Supplementary Information:**

The online version contains supplementary material available at (10.1186/s12864-022-08566-w).

## Background

Most cancer genome research studies have concentrated on the somatic aberrations that arise in the bulk tumor tissue. Much less care has been focused on the trajectory of change among single cancer cells and somatic cell evolution. Recent advance in high throughput single-cell DNA sequencing (scDNA-Seq) starts to making promising changes. scDNA-Seq dissects the mixture of normal and cancer tissues, thus affording an ultimate genomic resolution [1]. Through barcoding every single cell in sequencing, scDNA-seq provides profound evidence to decipher the intra-tumor heterogeneity (ITH) [2], recognize the rare cell population [3], and restore the evolutionary history of tumor cells [4, 5].

The heterogeneity in the single-cell tumor genome is from diverse aspects. The mainstream computational tools are tackled on detecting the profile of single nucleotide polymorphisms (SNPs), single nucleotide variations (SNVs), small insertion and deletions (Indels) [6–9], and copy number variations (CNVs) [10–12] for each tumor cell, and infer the phylogeny structure of tumor clones [13–16]. These tools’ performance may be nevertheless undervalued or overvalued, owing to the insufficient size of benchmarking data [17].

*In silicon* simulation is a cost-effective approach to generate as many scDNA-seq datasets as possible in a controlled manner to make reliable and valid benchmarking [18]. Currently, there is a collection of single-cell genome simulators (Table 1). CellCoal focuses on simulate SNVs with different somatic evolutionary trajectories [19]. Yu *et al.* developed SCSsim to produce SNVs, Indels, and CNVs, especially tackling the issue of allele dropout (ADO) and alleles unbalanced amplification frequently occurs in scDNA-seq [20]. SCSIM jointly mimics correlated single-cell and bulk DNA reads with SNVs [21]. Mallory *et al.* developed SingleCellCNABenchmark which generates *in silico* single-cell reads with CNVs [22]. However, existing tools do not offer the ground truth of CNV breakpoint and cell subclone label, which is highly required in downstream scDNA-Seq analysis [10–12].

**Table 1:** Overview of existing scDNA-Seq simulators

Tool	Journal	Supported Variants			Other Facilities	
		SNV	Indel	CNV	Cell Cluster	Breakpoints
CellCoal [19]	*Mol. Biol. Evol.*	$\checkmark $	-	-	-	-
SCSsim [20]	*Bioinformatics*	$\checkmark $	$\checkmark $	$\checkmark $	-	-
SCSIM [21]	*BMC Bioinformatics*	$\checkmark $	-	-	-	-
SingleCellCNABenchmark [22]	*PLoS Comput. Biol*	-	-	$\checkmark $	-	-
**SCSilicon**	-	$\checkmark $	$\checkmark $	$\checkmark $	$\checkmark $	$\checkmark $

This study proposes a new tool, SCSilicon, which efficiently generates single-cell *in silicon* DNA reads with minimum manual intervention. SCSilicon first creates the genome sequence (FASTA file) for each single-cell by automatically simulating a collection of genomic aberrations, including SNP, SNV, Indel, and CNV. Likewise, SCSilicon yields the ground truth of CNV segmentation breakpoints and subclone cell labels. Then, SCSilicon amplifies the genome and generates FASTQ reads. We have manually inspected a series of synthetic variations (SNP, SNV, Indel, and CNV breakpoint) generated by SCSilicon, and evaluated three start-of-the-art single-cell CNV callers.

## Implementation

### The SCSilicon framework

Currently, SCSilicon implements four different simulation models, named as ‘SNPSimulator’, ‘SNVSimulator’, ‘IndelSimulator’ and ‘CNVSimulator’. Each of them has its own assumptions but can be accessed through a consistent, easy-to-use interface. The detailed information on these simulators is described in the following sections.

**Fig. 1:**
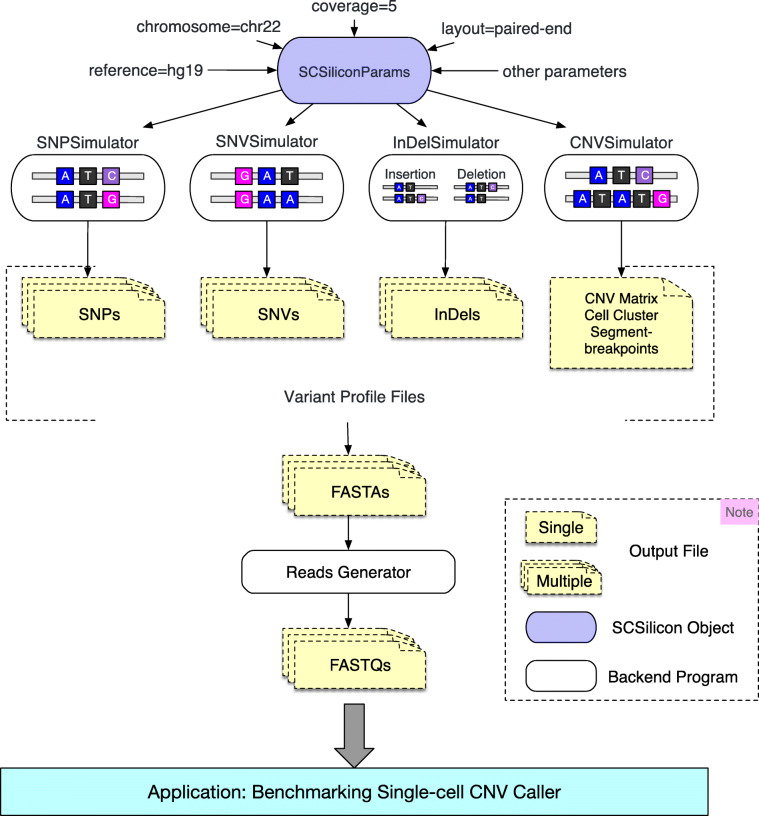
Illustration of SCSilicon framework

Figure 1 shows the overview architecture of the SCSilicon framework. The SCSilicon simulation process consists of two steps. The first step generates the parameters required for the following simulation process. The result of the first step is a parameters object named ‘SCSiliconParams’. The SCSiliconParams object is designed to store all the information required for a specific simulator, such as the reference genome version, the reads coverage, and the reads layout, etc. Users can change the default values of these parameters through the object member functions. The SCSiliconParams object allows different simulators to have their own parameters and provides flexibility for different simulation experiments.

For the second step, the SCSiliconParams object is passed to a specific simulator to generate a synthetic scDNA-seq dataset. As displayed in Fig. 1, firstly, the variant profile files are generated by a specific simulator according to the users’ parameters. Then, the mutated genome in FASTA format is generated by inserting various types of variations into the input reference sequence. Finally, the FASTA files are passed to the reads generator to generate scDNA-seq data in FASTQ format. SCSilicon uses a third tool, scssim [20], to generate the mutated genome and reads file.

### SNPSimulator

The SNPSimulator is to generate scDNA-seq data with SNPs. SCSilicon provides an interface to download the human dbSNP dataset from the UCSC genome browser automatically. For the single-cell simulation, a specific number of SNPs is selected from the human dbSNP dataset randomly and is inserted into the mutated genome to generate scDNA-seq data. The SNP number in a cell can be adjusted by the ‘snp_no’ parameter with a default value. SCSilicon also allows users to generate multiple cells once by the ‘cell_no’ parameter. For the multiple-cell simulation, 80% SNPs are shared by cells in one batch.

### SNVSimulator and IndelSimulator

Different from the SNPSimultor selecting SNPs from the dbSNP dataset, SNVSimulator generates the SNV profile file by randomly generating SNV sites from the reference sequence. Similarly, IndelSimulator generates the Indel profile file by randomly inserting or deleting reads with a length of 4 to 10 bps from the reference sequence.

### CNVSimulator

The CNVSimulator is designed to scDNA-seq data with CNVs and can be applied for the benchmarking of different single-cell callers. First, a CNV matrix that contains rows as cells and columns as bins is generated by CNVSimulator. The cell cluster number and the segment number of this CNV matrix can be adjusted by ‘cluster_no’ and ‘seg_no’ parameters, respectively. Then the CNV profile file and scDNA-seq data for each cell are generated according to the CNV matrix. CNVSimulator also outputs the cell clusters and segment breakpoints information for benchmarking purposes.

### Input and output

SCSilicon only needs users to enter the parameter configurations. Then, besides the sequence file (FASTQs format) for each cell, our SNPSimulator, SNVSimulator, InDelSimulator, and CNVSimulator also generates the ground-truth SNPs, SNVs, InDels, CNV matrix, cell cluster, segment-breakpoints as well. The detailed information for all variants, like rsid (Reference SNP ID), chromosome, position, reference alleles, copy number and etc. can be used for the ground-truth set for benchmarking variant callers.

## Results

### Visualization and inspection of genomics aberrations yielded by SCSilicon

We first applied BWA 0.7.17 [23] to align the synthetic single-cell FASTQ reads yielded by SCSilicon to the human reference (hg19). Then we visualized the ground-truth genomics aberration produced by SCSilicon and inspected whether the single-cell DNA reads carry the ground-truth abnormalities in SNP, SNV, Indel, and CNV, respectively.

**Fig. 2:**
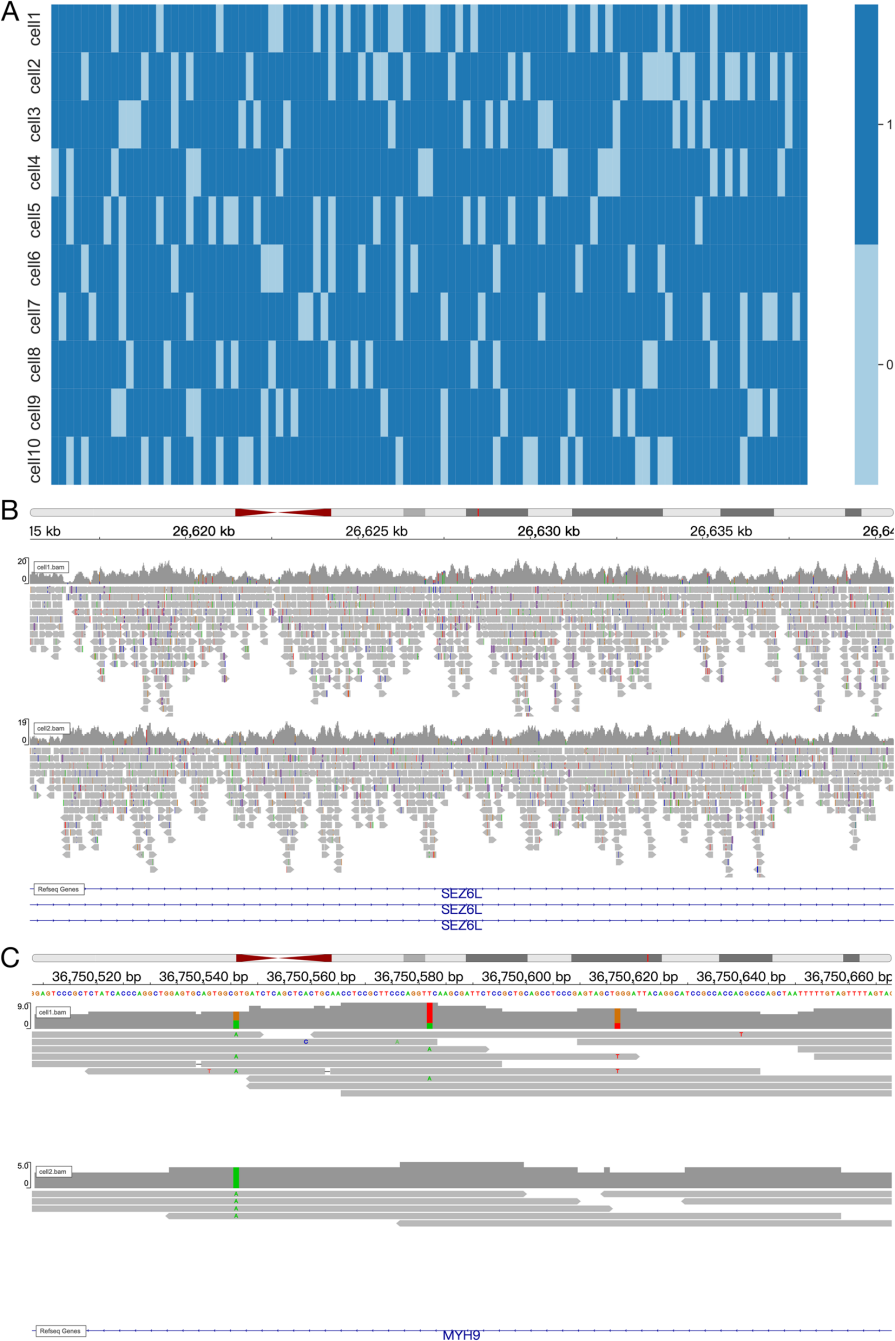
Visualization of simulated SNP. **A** Heatmap of random selected 100 SNPs across single-cells. 0 is reference allele, 1 is alternative allele. **B** IGV plot of SNP events. **C** IGV inspection of individual SNP events

Figure 2A exhibits the SNPs profile SCSilicon automatically generated across 10 single-cells. The cell population was assumed to share similar but slightly varied SNPs profiles. In Fig. 2A, we only visualized 100 randomly selected SNPs as we found when the number of SNP data increases (for example, 1000 SNPs), the heatmap would look a little fuzzy to clearly reflect the above characteristics.

Next, we leveraged IGV browser [24] to visualize the landscape of simulated SNPs across 25kb local genome region on gene *SEZ6L* (chr22:26,615,000-26,640,000) in two cells. As expected in Fig. 2B, the synthetic SNPs are randomly scattered in the reads. Likewise, cell 1 and cell 2 share alike but slightly different SNPs profiles. We then manually checked three SNPs (Fig. 2C). Located in chr22:36,750,551, SNP1 has three reference alleles G and three alternative alleles A in cell 1, and four alternative alleles A in cell 2. Located in chr22:36,750,587, SNP2 has seven reference alleles T and two alternative alleles A in cell 1, and five reference alleles T in cell 2. Located in chr22:36,750,622, SNP3 has five reference alleles G and two alternative alleles T in cell 1, and three reference alleles G in cell 2. Similarly, Additional file 1 Supplementary Fig. S1 and Fig. S2 demonstrates the SNV and Indel events SCSilicon generated. We also evaluated the generating-accuracy (the percentage of correctly generated SNPs, SNVs or Indels in sequence data from all SNPs, SNVs or Indels in ground truth data) of SCSilicon. We generated three dataset, SNP dataset, SNV dataset and Indel dataset respectively. Each dataset contained 10 cells and the average generating-accuracy was calculated for each dataset. The result show that the average generating-accuracies of these three dataset are all 100% which reflects the stability of SCSilicon.

**Fig. 3:**
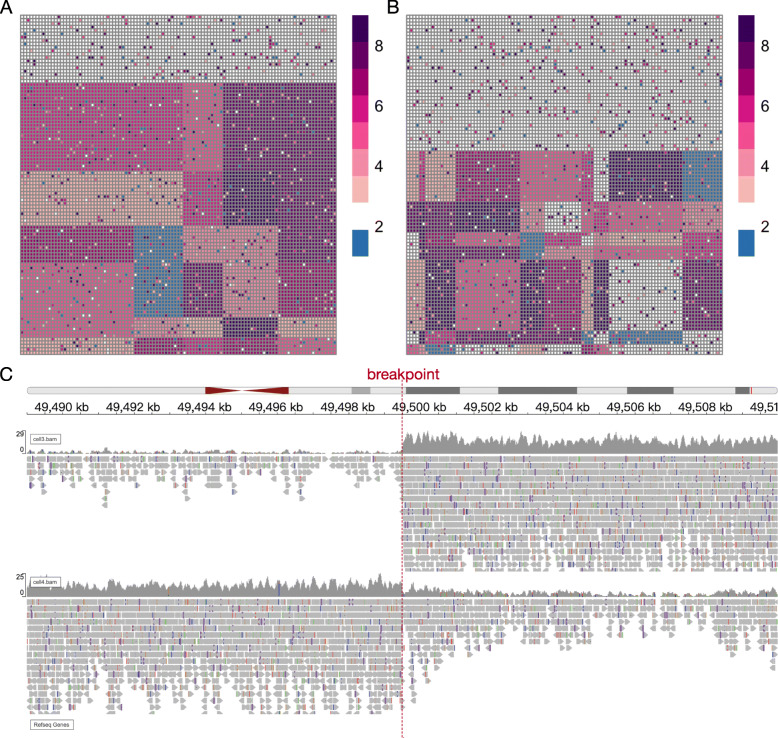
Visualization of simulated CNV. **A**-**B** Heatmap of two randomly generated CNV configuration across single-cells. The column and row represents the genome region bin and single cell, respectively. The value of the heatmap indicates the copy number, with blue, white, and red stands for copy number less than, equal to, and larger than 2, respectively. **C** IGV inspection of CNV breakpoint

Figure 3A and Fig. 3B are illustrations of two CNV matrices automatically generated by SCSilicon’s CNVSimulator with the random seed. The configuration is 100 single cells among chr22 with 50M as a bin, leading to matrices size of 100 × 70. The left-side matrix offers 20 normal cells and seven tumor cell clusters, with four CNV breakpoints and five CNV segments. The right-side matrix is more complicated. It owns 40% healthy cells and eight tumor subclones, with nine CNV breakpoints and ten CNV segments. Figure 3C is a snapshot of IGV visualization of CNV breakpoint chr22:49,500,000 across two cells. In cell 3, the breakpoint’s downstream region’s coverage is much higher than the upstream region. In cell 4, the breakpoint’s downstream region’s coverage is much lower than the upstream region. Meanwhile, the cell 3 breakpoint’s upstream region coverage is lower than the cell 4 breakpoint’s downstream region. These observations are concordant with the synthetic CNV ground-truth (cell 3 upstream region: 1, cell 3 downstream region: 8, cell 4 upstream region: 8, cell 4 downstream region: 3).

### Benchmarking state-of-the-art single-cell CNV caller

Recall that copy number variation (CNV) is considered to be a driving force in cancer progression and metastasis in single-cell genomics study [4, 5, 25]. Over the decades, an arsenal of scDNA-Seq CNV caller has been proposed. AneuFinder automatically qualifies the CNV profile leveraging a Hidden Markov model [10]. SCOPE [11] detected CNV by a Poisson latent factor model. SCYN [12] adopts SCOPE’s normalization policy and utilizing dynamic programming to conduct CNV segmentation. Be aware of each scDNA-seq CNV caller’s merits and demerits and choose the most robust one is essential to conduct single-cell genomics studies. Herein, we utilized the synthetic single-cell DNA reads generated by SCSilicon to evaluated three state-of-the-art CNV callers: AneuFinder, SCOPE, and SCYN.

**Fig. 4:**
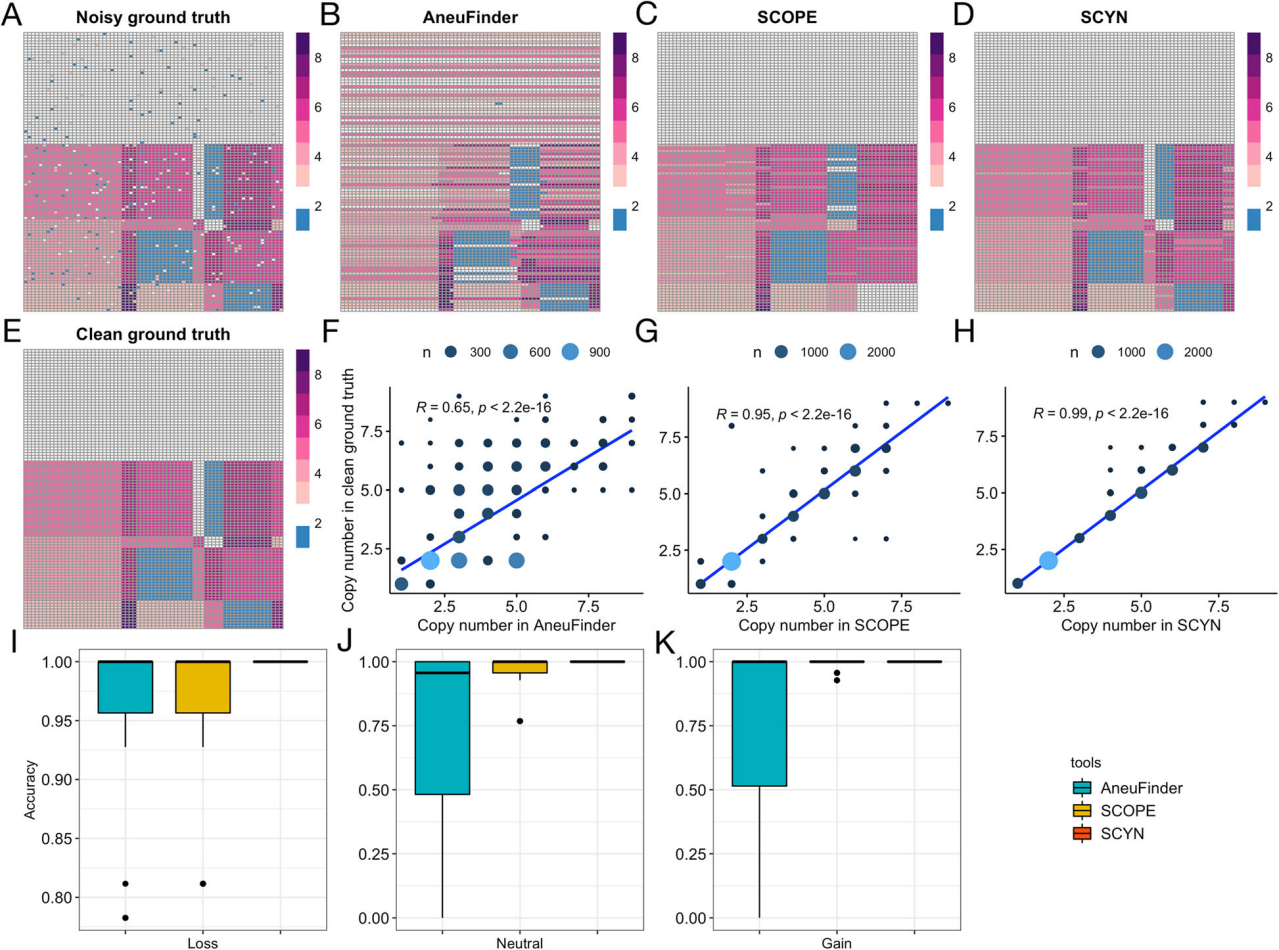
Benchmarking of single-cell CNV Caller for CNV dataset1. **A**-**E** CNV Heatmap of noisy ground-truth, AneuFinder, SCOPE, SCYN, and clean ground-truth, respectively. The column and row represents the genome region bin and single cell, respectively. The value of the heatmap indicates the copy number, with blue, white, and red stands for copy number less than, equal to, and larger than 2, respectively. **F**-**H** Scatter plot and Pearson correlation between clean ground-truth CNV and estimated CNV of AneuFinder, SCOPE, and SCYN, respectively. **I**-**K** The CNV calling accuracy on loss, neutral, and gain bins, respectively

We have mimicked two CNV matrices with 100 single cells on chr22 (50M bp/bin), dataset1 and dataset2 with noise rate 10% and 12%, respectively. For CNV dataset1, Fig. 4A and Fig. 4E displays the noisy and clean CNV ground-truth. This benchmark set has five cell subpopulations, with one normal cells clusters (average CNV is 2) and four tumor cell clusters with different CNV gains and losses. The ground-truth CNV matrix harbours six CNV breakpoints (chr22:29,500,000, chr22:31,500,000, chr22:39,000,000, chr22:40,500,000, chr22:43,000,000, chr22:49,500,000), leading to seven CNV segments. Figure 4B,C,D illustrates the estimated CNV matrix on the synthetic reads from AneuFinder, SCOPE, and SCYN, respectively. From bare-eye checking, SCOPE and SCYN can absorb the noise and distinguish the healthy cells, whereas AneuFinder’s performance is hugely skewed by the bias, mistakenly recognizing a large proportion of healthy cells as aneuploidy. However, AneuFinder successfully detected all six CNV breakpoints just like SCYN, while SCOPE attaches one fictional breakpoint between CNV segment chr22:29,500,000-31,500,000, and fails to call two vital breakpoints (chr22:40,500,000 and chr22:49,500,000). Furthermore, Fig. 4F,G,H reveals that the CNV inferred from SCYN has the highest Pearson correlation (*R*=0.99,*p*<2.2e^−16^) with ground truth CNV. We checked the CNV calling accuracy as well. In terms of neutral, gain, and loss, we treat them as three binary classification problems. We labeled the ground-truth and inferred CNV of each cell bin region with “neutral” (CN = 2) and “not neutral” (CN ≠ 2), “gain” (CN > 2) and “not gain” (CN ≤ 2), and “loss” (CN < 2) and “not loss” (CN ≥ 2). We defined the CNV calling accuracy as the correct predictions divided by the total number of predictions and used Python sklearn “metrics.accuracy_score” function to calculate it. Figure 4I and Additional file 1 Supplementary Table S1 demonstrates that SCYN manifests the highest CNV calling accuracy in neutral, gain, and loss region, respectively. For CNV dataset2 with a higher noise rate, Additional file 1 Supplementary Fig. S3 and Supplementary Table S2 demonstrate SCYN shows the highest Pearson correlation (*R*=0.96,*p*<2.2e^−16^) and the highest CNV calling accuracy in neutral, gain, and loss region respectively.

Overall, SCYN demonstrates the best efficacy in both breakpoint detection and CNV estimation, whereas AneuFinder has a deficiency in CNV normalization, and SCOPE is limited to correct breakpoint detection.

## Discussion

Simulation software is crucial in developing and validating the computational model for next-generation sequencing (NGS) data [18], so does it for single-cell genomics. To facilitate this necessity, we developed SCSilicon, a software tool that efficiently generates single-cell *in silicon* DNA reads with minimum manual intervention. SCSilicon automatically simulates a collection of genomic aberrations, including SNP, SNV, Indel, and CNV. Likewise, SCSilicon yields the ground truth of CNV segmentation breakpoints and subclone cell labels. We have manually inspected a series of synthetic variations (SNP, SNV, Indel, and CNV breakpoint) generated by SCSilicon. Furthermore, we assessed three state-of-the-art single-cell CNV callers AneuFinder, SCOPE, and SCYN. We discovered that SCYN demonstrated the best efficacy in both breakpoint detection and CNV estimation, whereas AneuFinder had a deficiency in CNV normalization, and SCOPE was limited on correct breakpoint detection.

Compared with existing single-cell genomics simulators, like SCSsim, SCSilicon has the following credits to highlight. (i) Our software is user-friendly. Users can easily install the package by PyPI management kit. SCSsim can only generate one cell at a time, while SCSilicon can generate a dataset that contains hundreds or thousands of cells with just a few lines of code (less than five lines). (ii) Our software provides more flexibility while it needs minor user intervention. SCSsim needs a user manual aberration configuration to generate the SNP, SNV, Indel, and CNV step by step. While in SCSilison, users can generate CNV datasets with different features by simply adjusting the parameter configuration at one time, like the percentage of normal cells, the number of cell clusters, the number of segments for one chromosome, and the rate of noise values. Then SCSilicon automatically generates all genomics aberration configurations to ease users from pre-processing. (iii) SCSilicon is useful for the benchmarking of single-cell CNV calling tools. Except for the sequence data of all cells in one dataset, SCSilicon also generates the ground truth CNV matrix, the detailed information of cell clusters and segments. The ground truth CNV matrix can be interactively visualized in scSVAS (https://sc.deepomics.org [26]). To our knowledge, no existing tools pay special attention to CNV segmentation breakpoints and subclone cell clusters. We output these two ground-truth information, providing a straightforward way to assess a CNV caller’s performance.

We plan to conduct several enhancements in the future. (i) Currently, SCSilicon simulated the SNPs, SNVs, and Indels through random sampling. We intend to create the point mutations based on an evolutionary model. In this way, SCSilicon can benchmark the SNV phylogeny callers in the future. (ii) As SCSilicon employs the API from SCSsim, the generated single-cell reads only fit the multiple annealing and looping-based amplification cycles (MALBAC) protocol. In the next step, we prepare to implement a Protocol Profiler to learn ADO and bias from diverse single-cell sequencing protocols, including MALBAC [27], degenerate-oligonucleotide-primed polymerase chain reaction (DOP-PCR) [28], Transposon Barcoded (TnBC) [29], and 10x [2].

## Conclusions

To conclude, we introduce a user-friendly single-cell DNA reads simulator, SCSilicon, which automatically creates a collection of genomic aberrations, including SNP, SNV, Indel, and CNV. Moreover, SCSilicon yields the ground truth of CNV segmentation breakpoints and subclone cell labels. We have manually inspected a series of synthetic variations. We assessed the state-of-the-art single-cell CNV callers and found SCYN was the most robust one.

## Availability and requirements

**Project name:** SCSilicon

**Project home page:** https://github.com/xikanfeng2/SCSilicon

**Operating system(s):** Platform independent

**Programming language:** Python

**Other requirements:** Python 3.6 or higher

**License:** MIT License

**Any restrictions to use by non-academics:** None

## Supplementary Information

**Additional file 1** The supplementary figures and tables.

## Data Availability

Source code of SCSilicon is available at https://github.com/xikanfeng2/SCSilicon.

## Abbreviations

scDNA-Seq: Single-Cell DNA-Sequencing
ITH: Intra-Tumor Heterogeneity
SNP: Single Nucleotide Polymorphism
SNV: Single Nucleotide Variation
Indel: Small Insertion and Deletion
CNV: Copy Number Variation
ADO: Allele Dropout
rsid: Reference SNP ID
