# Supplementary material for: SCSilicon: a tool for synthetic single-cell DNA sequencing data generation
Source: BMC Genomics. 2022 May 11;23(Suppl 4):359. doi: 10.1186/s12864-022-08566-w (PMC9092674; doi:10.1186/s12864-022-08566-w)
Supplement: Supplementary file 1 — Additional file 1 The supplementary figures and tables. [file 12864_2022_8566_MOESM1_ESM.pdf]

SCSilicon: a tool for synthetic single-cell DNA sequencing data generation
Supplementary File

Xikang Feng*, Lingxi Chen

February 9, 2022

Contents

1 Supplementary Figures **2**

2 Supplementary Tables **3**

1 Supplementary Figures

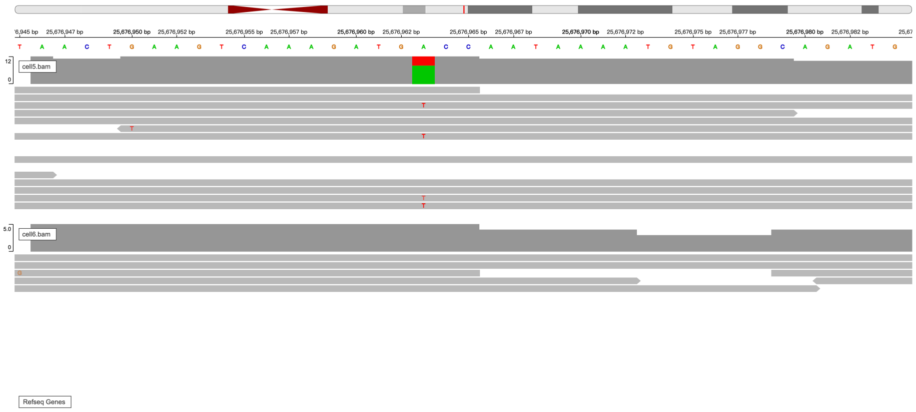

Figure S1: Visualization of simulated SNV IGV plot of SNV event on chr22:25676963.

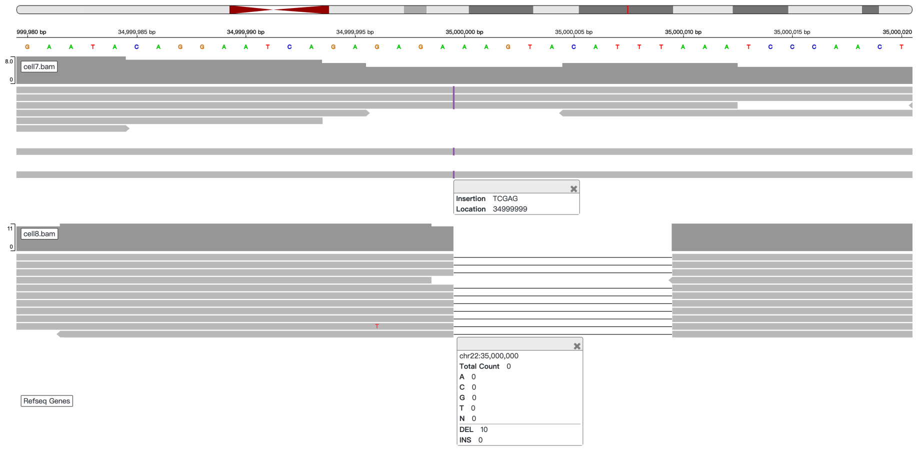

Figure S2: Visualization of simulated Indel IGV plot of Indel events.

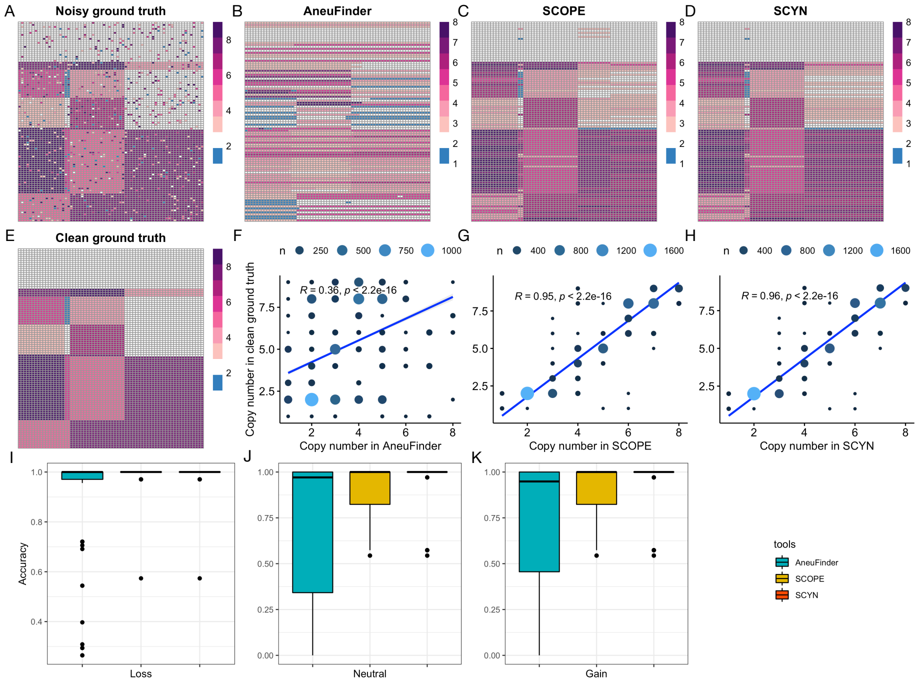

Figure S3: Benchmarking of single-cell CNV Caller for CNV dataset2. (A-E) CNV Heatmap of noisy ground-truth, AneuFinder, SCOPE, SCYN, and clean ground-truth, respectively. The column and row represents the genome region bin and single cell, respectively. The value of the heatmap indicates the copy number, with blue, white, and red stands for copy number less than, equal to, and larger than 2, respectively. (F-H) Scatter plot and Pearson correlation between clean ground-truth CNV and estimated CNV of AneuFinder, SCOPE, and SCYN, respectively. (I-K) The CNV calling accuracy on loss, neutral, and gain bins, respectively.

2 Supplementary Tables

Tool	Loss			Neutral			Gain		
	Q1	Median	Q3	Q1	Median	Q3	Q1	Median	Q3
AneuFinder	0.9565	1.0	1.0	0.4819	0.9565	1.0	0.5145	1.0	1.0
SCOPE	0.9565	1.0	1.0	0.9565	1.0	1.0	1.0	1.0	1.0
**SCYN**	1.0	1.0	1.0	1.0	1.0	1.0	1.0	1.0	1.0

Table S1: Benchmark for CNV calling accuracy in loss, neutral and gain region of CNV dataset1

Tool	Loss			Neutral			Gain		
	Q1	Median	Q3	Q1	Median	Q3	Q1	Median	Q3
AneuFinder	0.9706	1.0	1.0	0.3420	0.9706	1.0	0.4559	0.9485	1.0
SCOPE	1.0	1.0	1.0	0.8235	1.0	1.0	0.8235	1.0	1.0
**SCYN**	1.0	1.0	1.0	1.0	1.0	1.0	1.0	1.0	1.0

Table S2: Benchmark for CNV calling accuracy in loss, neutral and gain region of CNV dataset2
